# Reward-related neural correlates of early life stress in school-aged children

**DOI:** 10.1016/j.dcn.2021.100963

**Published:** 2021-05-15

**Authors:** Nicholas M. Morelli, Michael T. Liuzzi, Jacqueline B. Duong, Maria Kryza-Lacombe, Emma Chad-Friedman, Miguel T. Villodas, Lea R. Dougherty, Jillian Lee Wiggins

**Affiliations:** a San Diego State University, University of California, San Diego Joint Doctoral Program in Clinical Psychology, 6363 Alvarado Court, Suite 103, San Diego, CA, 92120, United States; b Department of Psychology, San Diego State University, 5500 Campanile Drive, San Diego, CA, 92182, United States; c Psychology Department, University of Maryland College Park, Biology/Psychology Building, 4094 Campus Drive, College Park, MD, 20742, United States

**Keywords:** Early life stress, fMRI, Reward, Connectivity

## Abstract

**Objectives:**

Early life stress likely contributes to dysfunction in neural reward processing systems. However, studies to date have focused almost exclusively on adolescents and adults, measured early life stress retrospectively, and have often failed to control for concurrent levels of stress. The current study examined the contribution of prospectively measured cumulative life stress in preschool-age children on reward-related neural activation and connectivity in school-age children.

**Methods:**

Children (*N* = 46) and caregivers reported children’s exposure to early life stress between birth and preschool age (mean = 4.8 years, *SD* = 0.80). At follow-up (mean age = 7.52 years, SD = .78), participants performed a child-friendly monetary incentive delay task during functional magnetic resonance imaging.

**Results:**

Children with higher levels of cumulative early life stress, controlling for concurrent stressful life events, exhibited aberrant patterns of neural activation and connectivity in reward- and emotion-related regions (e.g., prefrontal cortex, temporal pole, culmen), depending on the presence of a potential reward and whether or not the target was hit or missed.

**Conclusions:**

Findings suggest that stress exposure during early childhood may impact neural reward processing systems earlier in development than has previously been demonstrated. Understanding how early life stress relates to alterations in reward processing could guide earlier, more mechanistic interventions.

## Highlights

• Early life stress is associated with aberrant neural function.
• Altered neural function is apparent as young as early school age.
• Reward- and emotion-related regions show exaggerated activation/connectivity.
• Findings remain when controlling for concurrent stressful life events.

## 1 Introduction

Cumulative early life stress is a robust predictor of psychopathology across the lifespan (Carr et al., 2013; Kessler et al., 2010). Advances in functional neuroimaging have begun to shed light on the neurobiological mechanisms underlying this relationship, showing, for example that early life stress is associated with heightened amygdala response to negative emotional cues and reduced functional connectivity between amygdala and medial prefrontal cortex (Marusak et al., 2015; McCrory et al., 2011; McLaughlin and Lambert, 2017; McLaughlin et al., 2015). Prior studies also suggest that early life stress is related to decreased connectivity during emotion regulation paradigms (Burghy et al., 2012; Kim et al., 2013; Marusak et al., 2015). Importantly, a number of these neural alternations mediate the relationship between early life stress and psychopathology (Busso et al., 2017; Marusak et al., 2015), suggesting that neural processes play an important role in affecting outcomes following childhood stress exposure, and highlighting the need for continued investigation into other functional neural processes.

Reward processing may be an important neurobehavioral construct to target in relation to early life stress. Behaviors reflective of poor reward processing are linked to externalizing problems, risk-taking behaviors, and poor academic achievement (Lansford et al., 2017; Peeters et al., 2017; Watts et al., 2018). Functional magnetic resonance imaging (fMRI) studies have implicated reward-related neural abnormalities in multiple forms of psychopathology, including depression (Keren et al., 2018), anxiety (Dillon et al., 2014), substance use (Geier, 2013), psychosis (Radua et al., 2015), and bipolar disorder (Whitton et al., 2015). Importantly, early life stress has been linked to alterations in reward-related brain function, including hypoactivity in dopamine receptor-rich areas (e.g., ventral striatum) in response to emotional faces (Goff et al., 2013) and during gambling tasks with positive and negative feedback (Hanson et al., 2015a). However, only a handful of fMRI studies have employed specific reward processing paradigms (e.g., monetary incentive delay task) to demonstrate the relationship between early life stress and the individual components of reward processing, including the anticipation, receipt, and nonreceipt of the reward. This limited literature suggests that higher levels of early life stress are related to decreased activation in *anticipation* of rewards and increased activation upon *receipt* of rewards in reward- and emotion-related regions (e.g., striatum, caudate, putamen, amygdala, medial prefrontal cortex, dorsolateral prefrontal cortex, frontal cortex; Boecker et al., 2014; Dillon et al., 2009; Hanson et al., 2015a, 2015b; Mehta et al., 2010).

These studies provide important glimpses into the neural mechanisms of early life stress, yet several questions still remain. First, the few studies to date on early life stress and reward processing have focused exclusively on adolescent and adult reward processing. Second, with the exception of Hanson et al. (2015b), studies frequently do not control for concurrent life stress, weakening claims that early life stress plays a unique role in the development of reward processing neural circuitry. Finally, a major focus has been on the most severe forms of early life stress (e.g., child maltreatment), examined individually (i.e., physical, sexual, or emotional abuse; institutional neglect) in relation to brain structure/function. Yet, stressors that do not rise to the level of abuse – such as poverty, single-parenthood, parental depression or hostility – can substantially alter children’s developmental trajectories (Comeau and Boyle, 2018; M. Li et al., 2017; Sheidow et al., 2014). Parental depression, in particular, has received substantial attention as a predictor of children’s altered reward processing and subsequent psychopathology. From an early life stress perspective, depressed parents on average are less nurturing and more rejecting of their young children (Elgar et al., 2007). They are also less consistent in their administration of rewarding social cues and behaviors, such as reciprocity of facial expressions and praise (Aktar et al., 2017; Elgar et al., 2007). Such negative parenting behaviors can be experienced as a chronic developmental stressor for children who expect socially rewarding parental input. Simultaneously, parental depression, to a greater extent than other early life stressors, is thought to confer genetic risk for impaired reward processing. Evidence suggests that parental history of depression is associated with offspring’s aberrant reward processing and psychopathology irrespective of parenting behavior or children’s level of depressive symptoms (Olino et al., 2014; Wiggins et al., 2017). Therefore, parental depression appears influential in development of children’s reward processing abnormalities, through both early life stress and genetic neurobiological risk.

Moreover, although conceptualizing early life stress as individual forms of maltreatment can be fruitful to distinguish the effects of distinct types of adverse experiences (McLaughlin and Sheridan, 2016), there is also a robust body of literature supporting the importance of cumulative or additive stress exposure (Evans et al., 2013). Children exposed to early life stress rarely experience one form of adversity (Bethell et al., 2017; McLaughlin et al., 2010). Researchers have sought to address this issue in recent decades by modeling stress exposure cumulatively, operationalizing “risk” as the number of adversities experienced by an individual, rather than the severity or type of adversity. The concept of cumulative risk has obtained considerable traction within developmental science, with a robust body of literature now showing that exposure to multiple stressors consistently predicts more severe, adverse developmental outcomes compared to singular stress exposures (Evans et al., 2013). Among youth specifically, cumulative risk, relative to singular stress exposures, has been associated with poor academic achievement, impaired cognitive development, internalizing and externalizing psychopathology, criminality, and suicide (Appleyard et al., 2005; Crozier and Barth, 2005; Luster and McAdoo, 1994; Roberts et al., 2010; Savolainen et al., 2018). Only one study to our knowledge, by Boecker et al. (2014), incorporated a cumulative risk approach to investigate the association between early life stress and neural reward processing. However, reward processing was not assessed until adulthood. Research investigating the impact of cumulative early life stressors on reward processing during childhood will provide crucial and novel information about the developmental timing of stress-related disruptions in reward processing. Further, by including early life stressors that fall below the threshold for child maltreatment, this study will add insight into a broader population of children whose exposure to early life stress is pervasive but often undetected.

The current study examines the contribution of cumulative stressful life experiences measured in preschool-age children (Time 1; T1) on reward-related neural activation and connectivity in school-age children (Time 2; T2), controlling for concurrent, school-age life stress. In addition to activation, we examine brain connectivity using left and right amygdalae and ventral striata as seeds, given the demonstrated role of perturbations in brain networks, above and beyond single regions, in psychopathology (Dougherty et al., 2018). We hypothesized that youth with high compared to low levels of early life stress would demonstrate atypical patterns of activation, such as exaggerated activation differences between reward conditions, in emotion- and reward-related brain regions known to be recruited during reward processing (i.e., basal ganglia, amygdalae, frontal regions; Kumar et al., 2014; Oldham et al., 2018). Further, we expected that more vs. less early life stress would be related to altered striatum and amygdala connectivity during reward processing with similar emotion- and reward-related regions. Importantly, we expected that these effects would remain after controlling for concurrent stressful life events. By characterizing the neural substrates of reward processing in pre-adolescent populations, the current study advances understanding of the early developmental pathways from stress to manifested psychopathology.

## 2 Methods

### 2.1 Participants

Participants (mean age at T1 = 4.11, *SD* = 0.78) were recruited from a larger study (*N* = 175) at the University of Maryland aimed at investigating biological risk factors of depression (Dougherty et al., 2013). Participants were recruited via flyers and a commercial mailing list in the College Park, Maryland area. Parents with a lifetime history of depression were oversampled. The University of Maryland Institutional Review Board approved all study procedures. Parents provided parental permission for their children, and children 7 years and older provided assent. Exclusion criteria included any developmental or physical disability in the child, non-fluent English, and a lifetime history of a psychotic or bipolar disorder in either parent. Psychological assessment data are from two waves of assessment, first at preschool-age (3–5 years, T1), and approximately three years later (school-age, T2).

Neuroimaging data were acquired at school-age. Sixty-four children (5.9–9.6 years of age) underwent neuroimaging acquisition. Of those 64, one child was not scanned due to claustrophobia, 17 children were excluded because of issues with data collection (10 had incomplete scan data; 3 with excessive head motion (see criteria below); 2 completed a different scan protocol; one with a poor anatomical scan; one without behavioral data), for a final sample of *N* = 46 (see Table 1 for demographic information).

**Table 1:** Demographic and clinical characteristics.

Sex, % female	54.3 %
T1 Mean Age in Years (SD)	4.11 (0.78)
T1 Age Range	3.0−5.83
T2 Mean Age in Years (SD)	7.31 (0.72)
T2 Age Range	6.25−8.68
Race, N (%)	
White	24 (52.2 %)
African American	11 (23.9 %)
Asian	3 (6.5 %)
Multiracial	2 (4.3 %)
Other/Missing	6 (13.0 %)
Ethnicity, N (%)	
Hispanic	7 (15.2 %)
Non-Hispanic	37 (80.4 %)
Missing	2 (4.3 %)
T2 Child Depressive Symptoms Mean (SD)	4.55 (2.38)
T2 Child Anxiety Symptoms Mean (SD)	10.86 (5.64)
Early Life Stress Indicators, N (%)	
Parental Hostility >2 SD Above Mean	5 (10.9 %)
Single Parent Home	9 (19.6 %)
Family Income <$40,000	4 (8.7 %)
Neither Parent Attended College	11 (23.9 %)
> 4 Past 12 Month T1 Stressors (PAPA)	11 (23.9 %)
Exposure to Parental Depression	21 (45.7 %)
Concurrent Stressful Life Events	
Mean Count of Past 12 Month Stressors (PAPA) at T2	1.87 (1.41)

Note: T1 = Time 1 (preschool-age); T2 = Time 2 (school-age); SD = Standard Deviation; PAPA = Preschool Age Psychiatric Assessment; T2 Child Depressive and Anxiety Symptoms (parent-reported) were acquired from the PAPA.

The final sample did not differ significantly from the rest of the original 175 participants on sex, race, parental depression, parental education, or early life stress. None of the participants were taking psychotropic medications. Parents gave written informed consent, and child participants gave assent. Study procedures were approved by the University of Maryland’s Institutional Review Board.

### 2.2 Measures

#### 2.2.1 Early life stress

An early life stress index was calculated as a combination of several indices of stressors (see below for a description of each index; see Table 1 for descriptive statistics), each of which was dummy coded such that the presence of the stressor was coded as “1”. The stressors included (1) low family income (0=income >$40,000, 1=income< $40,000), (2) low parental education (0=at least one parent with a four-year college degree, 1=neither parent with a four-year college degree), (3) single parent household (0=absent, 1=present), (4) child exposure to parental depression (0 = no exposure, 1=exposure to parental depression from birth to T1), (5) high levels of observed parental hostility (0=hostility score less than 2 SD above the mean, 1=hostility score at least 2 SD above the mean), and (6) the experience of at least four additional non-overlapping stressful life events selected from Group A and B categories of the Preschool Age Psychiatric Assessment (PAPA; Egger and Angold, 2004; see description below) in the 12 months prior to the T1 assessment. These six indices were summed to create the Early Life Stress Index, with higher scores reflecting greater levels of early life stress. The stressors were chosen to reflect multiple distinct, yet related, aspects of the early rearing environment. See Chad-Friedman et al. (2021) for more details on the early life stress index. A similar cumulative measure of early life stress has been used elsewhere (Barch et al., 2018).

#### 2.2.2 Exposure to parental depression

The Structured Clinical Interview for DSM-IV (SCID-IV), Non-Patient version (First et al., 2002) was used to measure children’s exposure to parental depression. Each interview was conducted with the primary caregiver and the timing of parental depression was captured using a life-calendar approach, which was incorporated into the SCID-IV.

#### 2.2.3 Exposure to parental hostility

An observational parent-child interaction task based on a modified version of the Teaching Tasks Battery (Egeland et al., 1995) was used to assess parental hostility with the primary caregiver (95.2 % mothers). Parental hostility reflects a parent’s expression of anger, criticism, and frustration toward a child. The parental hostility paradigm included six developmentally appropriate tasks, which were rated on a 5-point scale (Cronbach alpha = 0.76; inter-rater reliability intraclass correlation coefficient [ICC] = 0.89, *n* = 38) and averaged to create a total parental hostility variable.

#### 2.2.4 Stressful life events

The sixth and final indicator of the early life stress index was operationalized as exposure to four or more additional, non-redundant stressful life events involving the child and family in the 12 months prior to the interview, assessed by the PAPA (Egger and Angold, 2004). The stressful life events for this indicator were selected from Group A and Group B categories of stressors in the PAPA and represented stressful life events experienced by children that were not captured by the other early life stress indicators, including moving, parental divorce, separation from parents, and changes in childcare or schooling (see supplementary materials for the full list of possible stressors). As with the other early life stress indicators, this indicator was defined dichotomously (0 = endorsement of three or fewer PAPA stressors, 1 = endorsement of four or more PAPA stressors), and was included to assess the presence of a high number of additional stressors. Given the focus of the present investigation, more severe, low-prevalence stressful life events such as physical and sexual abuse were not included. The interviews were conducted with the primary caregivers.

Parental hostility and the number of stressors were dichotomized to equally weight each stressor in the Early Life Stress index. Cut-off points were data-driven and chosen to indicate high and non-normative levels of hostility (>2 SD above the mean) and stressful life events (>4), which were present in <10 % of the study sample.

#### 2.2.5 Concurrent stressful life events

Parents completed the PAPA (Egger and Angold, 2004) again at T2, where they reported on stressful life events involving the child and family that occurred in the past 12 months (i.e., the same stressors assessed at T1).

#### 2.2.6 Child current anxiety and depressive symptoms

Parents reported on their children’s anxiety and depressive symptoms at T2 via the PAPA (Egger and Angold, 2004). Emotional disorders measured included anxiety (selective mutism, specific phobia, agoraphobia, separation anxiety disorder, generalized anxiety disorder, social phobia) and depression (dysthymic disorder, major depressive disorder, depression not otherwise specified). To maximize recall, symptoms occurring 3 months prior to T2 were rated. Total scores for the symptom scales were derived by summing all items that made up any anxiety disorder to create a total anxiety symptoms scale (ICC = .97, α = .81) and all items that made up any depressive disorder to create a total depressive symptoms scale (ICC = .89, α = .94).

#### 2.2.7 Reward processing task

During neuroimaging acquisition, participants completed a child-friendly version of a monetary incentive delay task (“piñata game”) previously shown to elicit reward-related brain activation (Helfinstein et al., 2013). Before beginning the game, all participants were informed that they could receive up to $15 depending on how well they performed the task. The piñata game was projected onto a screen in front of the MRI scanner and participants viewed it using a mirror attached to a head coil. Participants hit the piñata using a response-box inside the scanner. The piñata task consisted of a cue (2000 ms) period, during which the participants were informed if they could receive a reward for hitting the target (conditions: 50 % reward condition, 50 % no-reward). Regardless of condition, participants were told to try to hit the piñata. Across conditions, there was a varied anticipation period during which the participant waited to hit the target (2500−5500 ms). When the target dropped, participants either hit the target (breaking the piñata) or missed (piñata swung away; 1500 ms). There were four possible feedback scenarios: 1) reward/hit, 2) reward/miss, 3) no reward/hit, 4) no reward/miss. Time to hit the target was automatically adjusted in real time based on each participants’ performance to maintain an approximate 2/3 hit, 1/3 miss ratio. Because our goal was to equalize task performance for all participants via the real-time adjustment of task difficulty for each participant, task performance is not meaningful. Each run of piñata lasted 4 min and 52 s, and each participant completed 3 runs, for a total of 60 trials across all three runs. A more detailed description of the task, with illustrative figures, can be found elsewhere (Wiggins et al., 2017).

#### 2.2.8 Neuroimaging acquisition

Functional and anatomical brain images were collected using a 3.0 T Siemens MRI scanner with a 12-channel head coil. Blood oxygen level-dependent (BOLD) images were acquired as 36 contiguous axial slices parallel to the AC-PC line, with whole-brain coverage, utilizing an echo planar single-shot gradient echo pulse sequence (matrix size = 64 × 64, TR = 2000 ms, TE = 25 ms, flip angle = 70°, FOV = 192 mm, voxel size = 3 × 3x3, 438 images across all runs). High-resolution anatomical images (T1-weighted magnetization prepared rapid acquisition gradient echo [MPRAGE]) were acquired for anatomical localization and spatial normalization (176 1.0 mm sagittal sliced, flip angle = 9°, matrix size = 256 × 256, FOV = 250 mm, voxel size = 1 × 1 × 1 mm).

### 2.3 Analytic plan

#### 2.3.1 fMRI data preprocessing

Standard fMRI data preprocessing protocols were implemented using Analysis of Functional NeuroImages (AFNI; https://afni.nimh.nih.gov/afni), including slice-time correction, realignment of functional images, spatial smoothing specified at 4 mm, and nonlinear registration for spatial standardization to the Talairach template. Image volume pairs with a frame-wise displacement exceeding 1 mm were censored from participant-level analysis, and participants with mean framewise head displacement ≥ 0.30 mm or censoring of ≥ 35 % of TRs were excluded from all analyses (3 participants).

### 2.4 Data analysis

#### 2.4.1 Activation

For each participant, we estimated a general linear model with regressors of interest: Condition (“no reward” and “reward”) and Performance (“hits” and “misses”). The anticipation period (which contained “Condition”) was convolved with AFNI’s ‘dmBLOCK’ function over a variable duration. The feedback period (which included “Condition” and “Performance”) was convolved with ‘BLOCK’. For each individual participant, these analyses produced beta coefficients at each voxel for reward and no reward trials during the anticipation period as well as reward/hit, reward/miss, no reward/hit, and no reward/miss trials for the feedback period. Head motion (x, y, z, row, pitch, yaw) and baseline drift polynomials were included as nuisance regressors in these activation models as well as the subsequent individual level connectivity models.

#### 2.4.2 Connectivity

Generalized psychophysiological interaction (gPPI) analysis was used to calculate connectivity between a specific region of interest (i.e., a seed) and all other regions of the brain. We focused connectivity analyses on the feedback period given prior work implicating this phase of the monetary incentive delay task in this age range (Dougherty et al., 2018; Wiggins et al., 2017). Based on prior literature (Hanson et al., 2018; VanTieghem and Tottenham, 2018), the current study used left and right amygdala, and left and right ventral striatum as seed regions. Masks for these seeds were created using the Talairach daemon atlas in AFNI (left amygdala = 756 mm^3^; right amygdala = 972 mm^3^; left ventral striatum = 108 mm^3^; right ventral striatum = 108 mm^3^). The end product of these analyses was a set of voxel-wise images that represent connectivity (i.e., the correlation of different brain regions’ activation) between each of the four seed regions and the rest of the brain in each task condition (reward/hit, reward/miss, no-reward/hit, no-reward/miss).

#### 2.4.3 Second-level analyses

In order to analyze data across participants, we used AFNI’s 3dMVM program to create whole-brain ANCOVAs to identify the effect of early life stress on reward-related brain activity and connectivity. All models controlled for concurrent stressful life events. Analyses were run separately for anticipation and feedback conditions. In the anticipation model, early life stress was included as the quantitative between-subjects factor, while Condition (reward vs. no reward) was the within-subject factor. The contrast of interest was Early Life Stress x Condition, which evaluated the degree to which early life stress related to brain activation/connectivity, depending whether a reward was anticipated. In the feedback model, early life stress was included as the quantitative between-subjects factor, and Condition (reward vs. no reward) and Performance (hit vs. miss) were included as the within-subject factors. The contrast of interest for these models was Early Life Stress x Performance x Condition, which evaluated the degree to which early life stress related to brain activation/connectivity, depending on whether there was a potential reward and whether the target was hit or missed. Lower order terms were also included in the models.

Results were corrected for multiple comparisons using AFNI’s 3dClustsim for cluster correction, with the mixed-model spatial autocorrelation function (-acf) and the NN1 bisided option, to allow cluster positive and negative voxels separately. 3dClustsim used a group mask based on combining brain regions where 90 % of participants had valid data. The cluster threshold across all models was *k* ≥ 28 with a conservative height threshold of *p* < .005.

#### 2.4.4 Additional analyses

We completed additional analyses to evaluate the impact of other factors in our sample, including age, sex, residual head motion, depression, and anxiety at T2. For a targeted approach, values were extracted from clusters of interest, averaged, and analyses were re-run covarying for each additional factor. To examine the extent to which parental depression may have driven potential findings (e.g., through genetic risk; Elgar et al., 2007; Olino et al., 2014), we applied this same approach using an updated early life stress variable that omitted child exposure to parental depression as an indicator, controlling for parental lifetime report of depressive disorders separately.

## 3 Results

### 3.1 Participant characteristics

Table 1 summarizes participant characteristics. When controlling for concurrent stressful life events, early life stress was not significantly correlated to school-age depression (*r*=-.210, *p* = .283) or anxiety (*r* = .103, *p* = .602). Additionally, early life stress and concurrent stressful life events were not significantly related (*r* = .260, *p* = .081).

### 3.2 Whole-brain activation analyses

#### 3.2.1 Anticipation

##### 3.2.1.1 Early life stress x reward condition

**Fig. 1:**
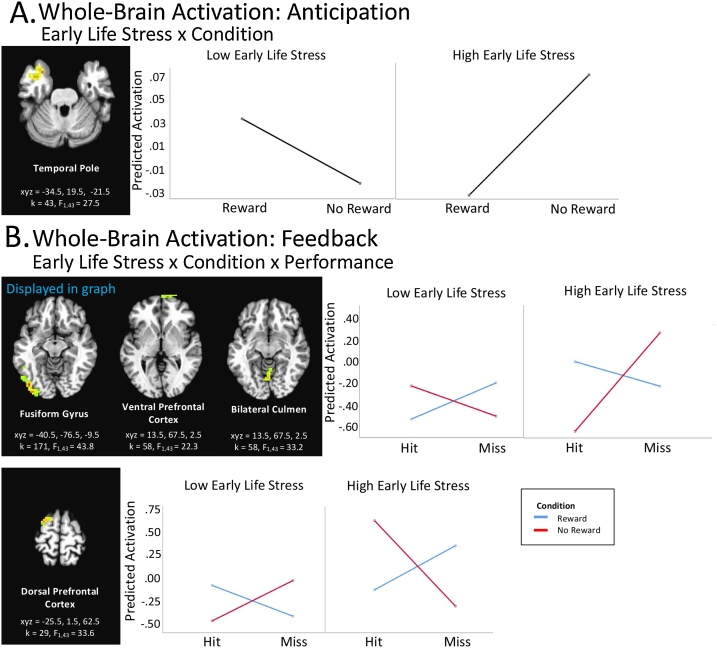
Activation Analyses. A) *Early Life Stress x Condition x Performance.* Graphs display the interaction between Early Life Stress, Condition (reward vs. no reward), and Performance (hit vs. miss). B) *Early Life Stress x Condition*. Graphs in B represent relationship between Early Life Stress and condition (reward vs. no reward). Across analyses, for illustrative purposes, graphs display predicted brain activation values for indicated clusters based on low and high scores in our sample (i.e., low=-4.38, high = 6.51). When multiple regions differed significantly in their activation during a single condition, the graph from one cluster was shown to illustrate the direction of effects. Brain regions represent axial sections (left = left) with threshold set at whole-brain FDR-corrected *p* < .05.

Whole-brain corrected analyses revealed a significant Early Life Stress x Reward Condition interaction in the left temporal pole (see Table 2; Fig. 1B). Here, children with higher vs. lower levels of early life stress, controlling for concurrent stressful life events, showed decreased activation when anticipating a potential reward, yet increased activation when not expecting a reward.

**Table 2:** Significant clusters resulting from whole brain analyses.

k	F_1, 43_	x	Y	z	BA	Region
**ACTIVATION: ANTICIPATION**						
***Reward Condition***						
152	37.2	17	−83	−7	17,18,19	Lingual Gyrus, Cuneus
132	38.6	−17	−89	−7	17,18,19	Lingual Gyrus, Cuneus
*Early Life Stress x Reward Condition						
**43**	**27.5**	**−35**	**20**	**−22**	38,21,28	**Temporal Pole**
**ACTIVATION: FEEDBACK**						
***Early Life Stress***						
56	28.7	59	−35	−16	21,20	Middle/Inferior Temporal Gyrus
49	24.1	−29	17	54	6,8	Dorsolateral prefrontal cortex
32	21.8	26	−71	−34	–	Pyramis, Uvula
***Concurrent Stressful Life Events***						
96	20.8	38	−44	33	40,39	Superior Temporal Gyrus, Precuneus, Inferior Parietal Lobule
***Reward Condition***						
2095	81	−23	−92	−7	18,19	Stratum, Occipital, Parietal, Fusiform, Angular, Parahippocampal Gyrus
81	19.8	5	17	6	–	Bilateral Caudate, Striatum
***Early Life Stress x Reward Condition***						
33	24.3	−53	−14	27	6,4,3	Pre/postcentral Gyrus
32	31.5	65	−11	−22	21,20	Inferior/ Middle Temporal Gyrus
***Concurrent Stressful Life Events x Reward Condition***						
65	21.1	59	−11	30	4,3,6,1,43,2	Pre/ Postcentral Gyrus
39	16	−38	−8	33	6,4,3	Pre/ Postcentral Gyrus
***Performance***						
137	22.3	−8	−53	18	31, 30, 23, 18, 19, 29, 18	Posterior Cingulate, Precuneus, Cuneus
137	31	8	5	51	32, 6, 24, 32, 8	Medial Prefrontal Cortex
123	21	32	−53	42	40, 7, 39, 19	Supramarginal Gyrus, Inferior Parietal Lobule
104	40.4	−32	−23	48	3,4,2,6,40	Post/ Precentral Gyrus
89	32.3	−5	−11	57	6, 31,24	Dorsomedial Prefrontal Cortex
87	24.6	41	−8	42	6,4,3	Dorsolateral Prefrontal Cortex
62	21.5	8	−53	−13	–	Bilateral Cerebellar Lingual
57	22.6	14	−5	9	–	Thalamus
53	17.8	8	−56	15	30,29,18,23,19	Posterior Cingulate
38	21.4	47	−29	18	41,40,13,42	Insula
38	14	−26	−77	27	7,31	Precuneus
36	15	38	23	27	9	Dorsolateral Prefrontal Cortex
35	19.1	−8	14	39	32,24	Medial Prefrontal Cortex
***Concurrent Stressful Life Events x Performance***						
30	17.3	−29	−14	7	–	Amygdala
***Condition x Performance***						
38	17.6	32	−62	−1	19,18	Fusiform Gyrus
31	17.8	32	47	−1	10	Ventral Prefrontal Cortex
29	17.7	14	−59	−13	–	Declive, Culmen
*Early Life Stress x Condition x Performance						
**171**	**43.8**	**−41**	**−77**	**−10**	18,19**,37,17,20**	**Fusiform Gyrus**
**58**	**22.3**	**14**	**68**	**3**	**10**	**Ventral Prefrontal Cortex**
**39**	**33.2**	**−2**	**−65**	**−7**	–	**Bilateral Culmen**
31	23.4	−26	−38	−37	–	Cerebellar Tonsil
29	24.6	−29	−62	−25	–	Culmen, Pyramis
**29**	**33.6**	**−26**	**2**	**63**	**6**	**Dorsal Prefrontal Cortex**
**LEFT AMYGDALA CONNECTIVITY: FEEDBACK**						
***Performance***						
28	15.7	35	−65	39	19,7,39	Precuneus
***Early Life Stress x Reward Condition x Performance***						
**48^**	**18.3**	**−23**	**−2**	**60**	**6**	**Dorsal Frontal Cortex**
**RIGHT AMYGDALA CONNECTIVITY: FEEDBACK**						
***Concurrent Stressful Life Events***						
57	18	26	−44	−34	–	Cerebellar Tonsil
41	16.9	−53	20	−13	9	Ventrolateral Prefrontal Cortex
*Early Life Stress x Reward Condition x Performance						
**100**	**17.1**	**−2**	**−29**	**57**	**6,5,3,4**	**Medial Frontal Gyrus**
**34^**	**14.9**	**−26**	**26**	**30**	**9**	**Lateral Prefrontal Cortex**
**LEFT VENTRAL STRIATUM CONNECTIVITY: FEEDBACK**						
***Performance***						
48	18.1	−11	17	51	6,8	Dorsal Medial Prefrontal Cortex
34	21.7	8	−26	45	31,5,24,6,7	Cingulate Gyrus
**RIGHT VENTRAL STRIATUM CONNECTIVITY: FEEDBACK**						
***Concurrent Stressful Life Events***						
53	20.1	−38	29	24	10,46,9	Lateral Prefrontal Cortex
41	17.4	−11	−17	−1	–	Thalamus
35	21.2	14	65	−1	10	Striatum
34	15.7	−20	5	9	–	Lentiform Nucleus
*Early Life Stress x Performance						
45	19.9	56	−32	−4	21	Temporal Parietal Junction

*Note*: Contrasts that did not yield significant clusters are not listed in this table. Clusters emerged from whole-brain analyses. * indicates a contrast of interest. Bolded clusters indicate clusters highlighted in figures. “^” indicates clusters that did not survive correction for depression.

#### 3.2.2 Feedback

##### 3.2.2.1 Early life stress x reward condition x performance

Early Life Stress x Reward Condition x Performance interactions were significant in multiple prefrontal and posterior regions (fusiform gyrus, ventral and dorsal prefrontal cortex, bilateral culmen, see Table 2; Fig. 1A). These interactions were driven by the no reward condition, in which children with higher levels of early life stress, controlling for concurrent stressful life events, exhibited exaggerated differences in activation to hit vs. miss conditions compared low early life stress children.

#### 3.2.3 Whole-brain connectivity analyses

##### 3.2.3.1 Right amygdala

###### 3.2.3.1.1 Early life stress x reward condition x performance

Whole-brain corrected analyses during the feedback period revealed a significant Early Life Stress x Reward Condition x Performance interaction for amygdala connectivity with two frontal regions (medial frontal gyrus and lateral prefrontal cortex). That is, in the reward condition, higher levels of early life stress, controlling for concurrent stressful life events, related to decreased right amygdala connectivity with frontal regions during hit trials but increased connectivity during miss trials. By contrast, in the no reward condition, the opposite pattern was detected: decreased connectivity during miss trials and increased connectivity during hit trials in relation to higher levels of early life stress.

##### 3.2.3.2 Left amygdala

###### 3.2.3.2.1 Early life stress x reward x performance

**Fig. 2:**
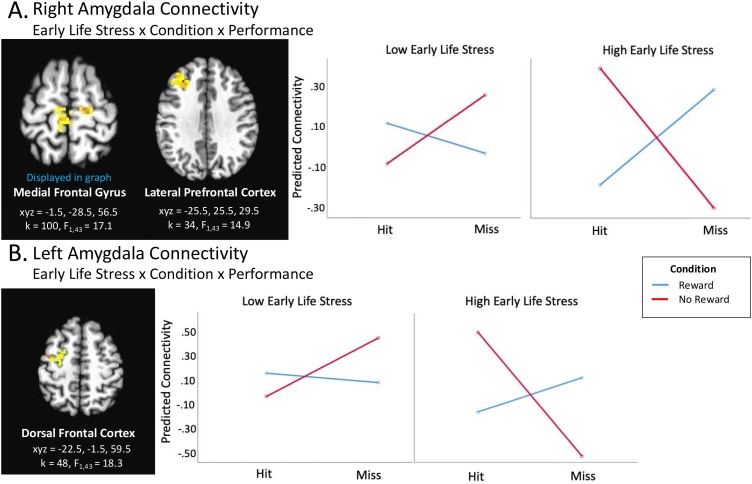
Connectivity Analyses: Feedback Period. A) Right Amygdala. *Early Life Stress x Condition x Performance.* Graphs display the interaction between Early Life Stress, Condition (reward vs. no reward), and Performance (hit vs. miss). B) *Early Life Stress x Condition x Performance.* Graphs in B represent the three-way interaction in the left middle frontal gyrus. Both left middle frontal gyrus clusters did not remain significant when covaried for depression; however, each showed a trend toward significance (indicated with a “^” in the Table 2; *p=*.069, right amygdala; *p* = .088, left amygdala). Across analyses, for illustrative purposes, graphs display predicted brain connectivity values for indicated clusters based on low and high scores in our sample (i.e., low=-4.38, high=6.51). When multiple regions differed significantly in their connectivity during a single condition, the graph from one cluster was shown to illustrate the direction of effects. Brain regions represent axial sections (left=left) with threshold set at whole-brain FDR-corrected *p* < .05.

Early Life Stress x Reward Condition x Performance interactions were significant for left amygdala connectivity with left dorsal frontal cortex (Fig. 2). This interaction was primarily driven by the no reward condition, in which higher levels of early life stress, controlling for concurrent stressful life events, were associated with greater differences in connectivity to hit vs. miss conditions, relative to lower levels of early life stress. Specifically, when there was no potential reward, higher levels of early life stress were linked to greater decreases in connectivity during miss trials but increased connectivity during hit trials.

Connectivity analyses using ventral striatum seeds yielded no significant clusters in contrasts of interest.

### 3.3 Additional Analyses

To summarize, all clusters showed the same pattern of results after covarying for age, sex, residual head motion, anxiety and depression symptoms, suggesting that our findings were not primarily driven by these potential confounders. However, for two clusters (dorsal frontal cortex for left amygdala as a seed, lateral prefrontal cortex for right amygdala as a seed), concurrent depression did have an impact. Both clusters showed a trend toward significance when adjusted for depression (indicated with a “^” in the Table 2; *p=*.069, right amygdala; *p* = .088, left amygdala); the direction of effects were similar with and without including depression in the model. When child exposure to parental depression was omitted from the early life stress index and parental lifetime depression history was controlled for separately, all clusters identified as significant in the original analyses remained significant, suggesting that parental depression/genetic risk was not the main driver of the results.

## 4 Discussion

To our knowledge, this is the first investigation of the effect of early life stress on subsequent reward-related brain function in children this young. Moreover, we address important gaps in the literature by (1) defining stress broadly (i.e., beyond the most extreme forms of early life stress), (2) including connectivity in addition to activation in our analyses, and (3) controlling for concurrent stressful life events. We found that children exposed to more versus less stress during the birth-to-preschool age period exhibited altered activation in brain regions associated with reward processing and emotion regulation (i.e., left superior temporal gyrus, frontal lobe regions) at school-age, as well as aberrant patterns of connectivity between amygdalae and temporal and frontal regions, depending whether there was a potential reward and whether the target was hit or missed.

These findings represent an important contribution to the literature, particularly in the context of cumulative risk, in which the sum total number of stressors experienced by an individual is posited to be more impactful than any one type of stressor (Evans et al., 2013). Cumulative stress, relative to single-risk or single-event factors, may have a particular impact on our understanding of neural reward processing across development. Previous literature suggests, for example, that single-event modeling, compared to a cumulative approach, has weaker predictive ability of negative developmental outcomes (Evans, 2003; Greenberg et al., 1999). Cumulative stress is also associated with poor developmental outcomes across a wide range of domains (e.g., cognitive, academic, socioemotional, behavioral), suggesting that several explanatory mechanisms may be at play. Within the cumulative risk literature, disruption of stress-response systems (i.e., allostatic load) is often posited as the primary underlying mechanism through which early life stress increases risk for poor outcomes (McEwen, 2012; McLaughlin and Sheridan, 2016). Our findings provide evidence that reward processing may be an additional transdiagnostic mechanism on which to focus, laying the foundation for future work to examine whether and how reward processing might mediate the effects of early life stress on youth’s developmental outcomes. Only one study to our knowledge has used a cumulative index of early life stress to predict neural correlates of reward processing (Boecker et al., 2014), though reward processing in that investigation was not assessed until adulthood. Our findings add to the cumulative risk theory by suggesting that the accumulation of life stressors may begin to alter key emotional and cognitive developmental processes (e.g., reward processing) at an earlier age than was previously known. Whereas a large body of work has supported the link between cumulative stress and various behavioral and socioemotional outcomes in early childhood (Evans et al., 2013), the present investigation is the first to identify associations between cumulative stress and reward-related brain function in children this young.

Consistent with prior literature (Boecker et al., 2014; Dillon et al., 2009; Mehta et al., 2010), more early life stress was associated with lower levels of activation in anticipation of a reward. Early life stress may reflect an environment with diminished rewards and/or unpredictable rewards/punishments (e.g., positive vs. negative interactions with parents, peers, and others; material resources at school, home, or in the neighborhood; exposure to danger, violence) (Belsky et al., 2012). Although speculative, it is possible that the types of alterations found in the present study could reflect a neurobiological response by children whose early environments were characterized by infrequent or unpredictable rewards (Novick et al., 2018). For example, children with parents who are depressed and/or rely on hostile parenting strategies may fail to predict displays of parental warmth. Of note, we observed the same pattern of results when removing child exposure to parental depression as an indicator of early life stress and controlling for it separately, reducing the plausibility that the associations identified here were driven purely by parents’ transmission of genetic risk. Similarly, parents in low-income families may not be able to consistently provide children with material rewards for good behavior. Through repeated failure to predict the occurrence of rewarding stimuli, synapses in key emotion- and reward-related brain regions may be pruned earlier and/or at a higher rate (Novick et al., 2018) and thus lead to the lower level of activation seen in the current study. In contrast to prior work, which found higher levels of activation across feedback conditions (Boecker et al., 2014; Dillon et al., 2009; Hanson et al., 2015b; Mehta et al., 2010), we found that levels of activation depended on whether a reward was available and whether the child hit or missed the target. Indeed, these results serve to highlight the importance of the reward context and the specificity of the alterations to the reward conditions. Similarly, prior studies observed lower levels of connectivity, in general, among children with high versus low levels of early life stress in the general context of a reward task (Hanson et al., 2018). Yet, again, our findings revealed more nuance in the relationship between early life stress and patterns of connectivity, i.e., connectivity levels depend on whether a reward was available and whether the child hit or missed the target.

The most consistent finding, in every cluster, was that children with higher early life stress demonstrated more extreme *differences* in activation and connectivity across the reward and performance conditions, often in the opposite direction, compared to children with lower early life stress. Given that a number of significant clusters were observed in networks involved in emotion regulation (e.g., temporal pole, superior and middle frontal regions) these results may reflect the impact of early life stress on children’s ability to appropriately modulate emotions following both reward receipt and nonreceipt. In particular, children with high early life stress demonstrated strong differences in activation and connectivity between target hits and misses in the *absence* of a potential reward. It is possible that children with lower levels of early life stress are able to respond accurately to the low-stakes nature of a no-reward condition, whereas children with high levels of early life stress are unable to appropriately regulate their emotional reactions even in these low-stakes situations. Future studies could probe this possibility by incorporating concurrent behavioral or self-report measures that assess children’s subjective emotional states during imaging of reward-related tasks.

Interestingly, in addition to our findings in reward and emotion regulation regions, the same pattern of exaggerated activation differences in children with higher levels of early life stress was also observed in visual regions (i.e., left superior occipital gyrus). This was not expected, and it is not immediately clear how or why early life stress would be related to reward-related activation in visual areas. The monetary incentive delay task requires rapidly pressing a button when a target appears, and thus places particularly strong demands on visuospatial attention. One possibility is that children with relatively low levels of early life stress were able to consistently modulate their visual attention, regardless of whether there was a potential reward and whether the target was hit or missed, while those with levels of early life stress, more influenced by the different reward conditions, did not show this attentional control. This interpretation would align with existing literature demonstrating alterations in visual attention to emotion and reward cues among children exposed to adversity (Birn et al., 2017; Kumar et al., 2014), as well as a handful of studies that have found unexpected reward-related activation in visual regions (Boecker et al., 2014; Silverman et al., 2015). Although it is difficult to ascribe specific functions to individual activated regions, as brain function typically involves coordinated networks of activity, our findings suggest that additional work may investigate the potentially important role of visual region activation in the relationship between early life stress and psychopathology.

We provide novel, preliminary evidence that the cascading neural effects of early life stress may begin to take hold as early as six- and seven-years old, altering core reward-related affective and cognitive processes that are known to confer risk for later psychopathology (Novick et al., 2018). This has important implications for treatment and prevention strategies. First, our findings justify the implementation of interventions and services for parents of young children that address forms of stress that are excluded from current definitions of trauma and child maltreatment. Directing resources toward vulnerable families (e.g., in low SES or single-parent households in which harsh parenting and/or parental mental health issues are indicated) could attenuate the negative impact of early life stress on children’s later neural reward processing. Such resources could include parenting classes that provide more positive alternatives to harsh parenting, opportunities for parental education advancement, and basic economic assistance. Similarly, interventions in middle childhood that target reward processing deficits (i.e., subclinical anhedonia) or impairments in reinforcement learning through cognitive remediation techniques may be effective in reducing or even preventing adolescent psychopathology, presenting a potentially fruitful research direction based on this work (Garland, 2016; X. Li et al., 2016). Our findings lay the foundation for treatments that address the basic, dimensional components of psychopathology (e.g., aberrant reward processing), rather than the presenting psychiatric disorders later on (Insel, 2014), potentially curbing the long-term negative impacts of early life stress on youth’s socioemotional development.

This study is not without limitations. First, our sample size was modest (*N* = 46). Our N is greater than previous reward processing and early life stress studies (n = 13 previously maltreated adults, n = 12 previously deprived adolescents; Dillon et al., 2009; Mehta et al., 2010) and represents a challenging-to-scan population (young children); nevertheless, the present study will require replication with larger samples. Relatedly, our study was underpowered to examine the unique contribution of specific early life stressors, in addition to their cumulative impact, on school-aged reward processing. Third, although the current study used a longitudinal design, neuroimaging occurred only at the school-age follow-up, precluding our ability to (1) determine whether the reward-related abnormalities in brain function identified here were present earlier in development and (2) formally test reward-related brain function as a mediator in the relationship between early life stress and later psychopathology. Fourth, although the cumulative risk approach has proved widely useful for highlighting the significance of multiple versus single childhood adversities, others have criticized its failure to distinguish between distinct types of environmental experience, the fact that individual stressors likely vary in the magnitude of their impact, and the implicit assumption that all forms of early life stress influence development through the same underlying mechanisms (McLaughlin and Sheridan, 2016). Future studies with larger samples sizes should probe distinct dimension of early life stress (e.g., experiences of deprivation versus threat; McLaughlin and Sheridan, 2016; Sheridan and McLaughlin, 2014) and their specific associations with neural correlates of reward processing.

Despite these limitations, the present study has several strengths. Most notably, fMRI scans occurred when participants were 6–9 years old. As such, the current sample is substantially younger than that of any other previous study to examine the relationship between early life stress and reward processing. In addition, early life stress in the current study was assessed prospectively and defined broadly as experiences of parental hostility, single-parenthood, low-parental education, low income, family stress, and parental depression. Although it is important to understand the impact of child abuse and neglect on neurodevelopment, the current definition of early life stress captures experiences that are much more common and that frequently co-occur among the general population of preschoolers. Lastly, we were able to take concurrent stressful life events into account, increasing the likelihood that neural effects were specific to early life stress.

These findings, which demonstrate that early life stress is associated with aberrant patterns of activation and connectivity in childhood above and beyond concurrent stressful life events, add crucial knowledge to our understanding of the potential neurodevelopmental pathways through which early stressful environments lead to the emergence of psychopathology. This study also lays the foundation for future work that formally examines children’s aberrant reward processing as a mediator in the relationship between early life stress and of subsequent psychopathology, a finding that has been demonstrated in samples of adolescents but not younger children as of yet (Hanson et al., 2015b). Clinically, these findings justify treatments that target core mechanisms of psychopathology, potentially preventing the emergence of anxiety, depression, and other psychiatric disorders related to reward system dysfunction.

## Funding information

This research was supported by the Maryland Neuroimaging Center Seed Grant Program (LRD), 10.13039/100000001National Science Foundation in partnership with the University of Maryland Type: ADVANCE Program for Inclusive Excellence (L. Dougherty & T. Riggins), University of Maryland College of Behavioral and Social Sciences Dean’s MRI Research Initiative RFP Program (L. Dougherty & T. Riggins), Behavioral and Social Sciences Dean’s Research Initiative (L. Dougherty), and the Research and Scholarship Award (L. Dougherty). This research was also supported by a NARSAD Young Investigator Award#26802 (J.L. Wiggins).

## Declaration of Competing Interest

The authors report no declarations of interest.
