# Supplementary material for: Reward-related neural correlates of early life stress in school-aged children
Source: Dev Cogn Neurosci. 2021 May 15;49:100963. doi: 10.1016/j.dcn.2021.100963 (PMC8144345; doi:10.1016/j.dcn.2021.100963)
Supplement: Supplementary file 1 [file mmc1.docx]

**Supplementary Material:**

**List of Preschool Age Psychiatric Assessment (PAPA) stressors considered for indicator #6 of the early life stress index**

- Parental separation
- Parental divorce
- New parental figure moved into the home
- Child moved
- Child changed school
- Move resulted in end of a close relationship
- Death of a pet
- Noticeable reductions of family standard of living
- Loss of home
- Parental arrest
- Parental hospitalization
- Child separation from parent
- Child involved in serious car accident
- Child struck by car/bike
- Child poisoning
- Child burned
- Child near drowning
- Child serious fall
- Serious attack by animal
- Fracture of child’s bones
- Child diagnosed with serious illness
- Child hospitalized
- Death of someone close to the child
- Natural disaster
- Fire
- Child exposure to event that could cause death/severe injury
- Victim of physical violence
- Removal from home
- Illness/injury of someone close to the child
